# Low-Cost Integrated Optical Microscope and Contact-Mode Atomic Force Microscope System Based on DVD Optical Pickup Unit

**DOI:** 10.3390/s26103170

**Published:** 2026-05-17

**Authors:** Likang Wang, Chenyu Dong, Yufan Jin, Zhenda Lu, Yan-Qing Lu, Weihua Zhang

**Affiliations:** 1 College of Engineering and Applied Sciences, Nanjing University, Nanjing 210023, China; 2 State Key Laboratory of Analytical Chemistry for Life Science, Nanjing University, Nanjing 210023, China; 3 MOE Key Laboratory of Intelligent Optical Sensing and Manipulation, Nanjing University, Nanjing 210023, China

**Keywords:** optical pickup head, contact-mode, atomic force microscope, optical imaging

## Abstract

**Highlights:**

**What are the main findings?**

- A low-cost, modular integrated optical and atomic force microscope based on a commercial optical pickup unit is developed, realizing stable contact-mode imaging with signal noise below 2 nm.
- By exploiting the inherent fixed conjugate planes within the optical pickup unit, the system successfully overcomes unfixed focal plane issues, enabling real-time optical tracking of the probe and sample surface at a 1.5 μm resolution.

**What are the implications of the main findings?**

- Constructed from off-the-shelf optomechanical parts and 3D-printed components, this system provides a highly accessible, easy-to-assemble, and expandable atomic force microscope hardware solution for both scientific research and education.
- The integrated optomechanical architecture with real-time visual positioning significantly simplifies tip-sample regulation, offering more intuitive and reliable support for contact-mode applications.

**Abstract:**

We design and implement a low-cost, modular integrated optical and atomic force microscope (AFM) based on an optical pickup unit (OPU) capable of stable contact-mode operation. By exploiting the inherent fixed conjugate planes within the OPU, we overcome the imaging difficulties caused by unfixed focal planes. This allows for real-time optical observation of the AFM probe position and the relative tip-sample position during operation with an optical resolution of 1.5 μm. Furthermore, by optimizing the circuit design and scanning logic, we suppress the OPU lens drift and system noise on imaging, enabling stable contact-mode operation with a signal noise of less than 2 nm. Built with off-the-shelf low-cost mechanical and electronic components alongside a few custom 3D-printed parts, this system features low cost, easy assembly, and high expandability.

## 1. Introduction

The rapid development of consumer electronics has provided various high-performance, low-cost optoelectronic components, making the realization of low-cost, DIY high-end scientific equipment possible [1,2,3]. One example is the AFM based on a DVD OPU module. Utilizing the OPU—a highly integrated, high-precision, and high-sensitivity optical distance reading device—researchers pioneered the demonstration of OPU-based AFMs [4,5]. This breakthrough sparked widespread interest in research and DIY communities, leading to extensive efforts to expand this system from various angles. Wang et al. first proposed a quad-rod actuation structure driven by piezoelectric disk buzzers and established a compact AFM system utilizing a commercial optical pickup unit for cantilever deflection detection, which provides a mature, low-cost hardware framework for the open-source AFM community [6]. Unger et al. developed a compact, low-cost AFM based on an optical pickup head and introduced a flexure structure to suppress ground vibration and improve imaging stability [7]. These efforts have significantly improved the reliability, usability, and precision of the system, paving the way for the broad application of OPU-based AFMs in education and scientific research [8,9].

However, in practice, the high integration of the OPU causes difficulties in system design and operation. The OPU can be viewed as a highly integrated confocal microscope with a built-in tunable focusing objective of short working distance [10]. To minimize size, commercial OPUs use a beam splitter (BS) to fold the optical path between the tube lens and the detector. This folded optical path design makes it challenging to use an external microscope imaging system to closely observe the tip position, the sample surface, and the tip-sample relative position in real-time through the beam splitter [11,12], especially during the approach and region-selection processes. Meanwhile, equipped with a high numerical aperture (NA) objective, the OPU naturally enjoys superior optical imaging capabilities; researchers have already leveraged OPUs to demonstrate high-quality biological imaging, 3D optical inspection, and even nanolithography systems [13,14,15,16]. It is therefore interesting to find a way to integrate the optical imaging capability of the OPU into an AFM system.

Furthermore, to achieve better integration and lower cost, the component selection within OPUs differs significantly from that of performance-first scientific instruments, which introduce engineering limitations and challenges. As consumer-grade mass-produced devices, OPU modules are designed to prioritize mass production compatibility and cost control rather than the extreme electrical noise suppression and long-term operational stability required for high-precision nanoscale detection. This results in unavoidable inherent limitations in their internal circuits and mechanical structures when applied to precision detection scenarios such as AFM. For instance, the performance of the built-in laser diode (LD) driving circuit often suffers from high noise levels, being susceptible to temperature drift, supply voltage fluctuation, and device aging [17]. Additionally, utilizing a voice coil motor (VCM) to control the lens inherently impacts long-term system stability [18,19]. As an open-loop actuator, a VCM is originally designed for fast focusing rather than precise static positioning. Its mechanical hysteresis and creep effect are prominent under long-duration operation, which easily introduces subtle lens offset and degrades nanoscale imaging consistency. To mitigate these issues, previous researchers introduced tapping mode, employing signal modulation and demodulation to filter out irrelevant noise and system drift [20]. However, tapping mode requires sophisticated high-speed signal processing circuits to realize real-time modulation, demodulation, and phase lock of the cantilever vibration signal. In addition, this approach severely limits the application of OPU-based AFMs in fields that fundamentally require a contact mode (e.g., nanomechanical measurements [21,22] and probe direct-writing [23,24], to name a few).

Today, the above challenges have collectively limited the translation of OPU-based AFMs from proof-of-concept prototypes to practical, widely usable tools. For educational and DIY scenarios, intuitive visual guidance and stable contact-mode imaging are not optional but essential features that lower the barrier to operation and ensure experimental reproducibility. Most existing low-cost setups still rely on complex manual adjustment and offer little flexibility for on-demand modification, which greatly raises the learning curve for new users. Meanwhile, the absence of real-time visual feedback increases the risk of probe damage and inaccurate positioning, further compromising the reliability and consistency of measurement results. To address the above issues, we design an AFM based on a conjugate plane imaging architecture and comprehensively optimize the system’s stability and driving circuits, ultimately achieving stable contact-mode scanning. This AFM can be constructed entirely from standard commercial optomechanical components and a minimal number of 3D-printed parts, ensuring it is low-cost, easy to assemble, and highly expandable.

## 2. Principle and Design of the Integrated Optical Microscope and AFM System

**Figure 1:**
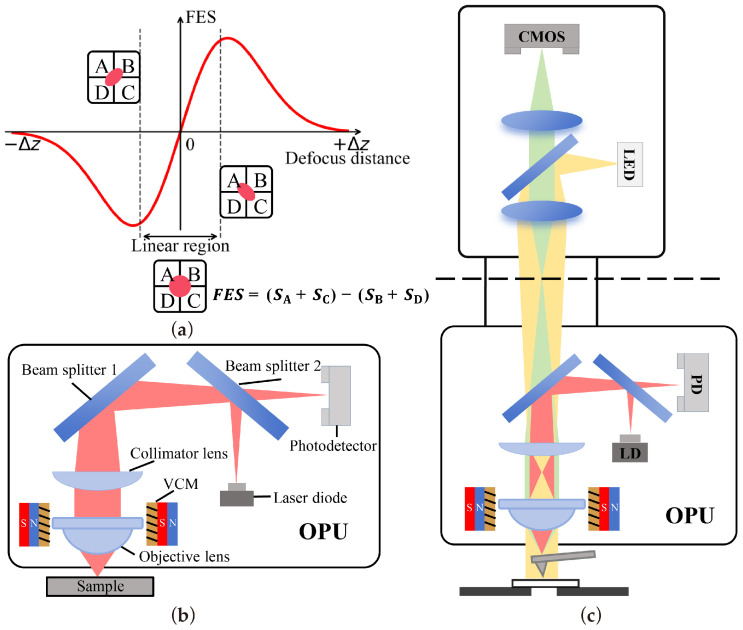
(**a**) Distance sensing principle of the OPU. (**b**) Structure of the OPU. (**c**) Design principle of the integrated OPU-based AFM and optical microscopy system. The dashed line labels the conjugate plane.

Figure 1a illustrates the fundamental principle of the OPU-based AFM. The laser beam emitted by the LD is focused on the sample surface via a collimating lens and an objective lens. The reflected laser beam successively passes through two beam splitters and irradiates the photodetector-integrated circuit (PDIC). As depicted, a distance variation in the measured object in the z-direction induces a shape change in the laser spot on the PDIC, which can be precisely quantified by the focus error signal (FES), calculated as:

(1) $$FES=(S_{A}+S_{C})-(S_{B}+S_{D})$$

where *S*_A_, *S*_B_, *S*_C_, and *S*_D_ denote the output voltage signals of the four respective quadrants of the PDIC. When the sample is perfectly positioned at the focal plane of the objective lens, the beam spot is circular, yielding an FES of zero.

To compress its physical footprint, the OPU employs a beam splitter to bend the optical path (Figure 1b). Because this beam folding occurs after the tube lens, the optical signal emanating from the focal plane is no longer collimated when it traverses the beam splitter. Consequently, it cannot be directly coupled to a standard infinity-corrected imaging system to observe the probe or sample, introducing significant experimental hurdles. More critically, since the OPU’s objective lens is mounted on a movable VCM, tracking and imaging the dynamically moving probe becomes a severe challenge.

Interestingly, the OPU system itself can be functionally regarded as a confocal system. The focal plane of the objective lens and the planes of the LD and PD are perpetually conjugate. Consequently, they remain conjugate with the image of the LD and PD on the opposite side of the beam splitter (the upper end of the OPU, dashed line in Figure 1c). Therefore, regardless of the objective’s dynamic z-position, it will always form an image on the plane conjugate to the LD at the upper end of the beam splitter. Based on this principle, if we image the conjugate focal plane above the beam splitter, we can continuously maintain in-focus imaging of the OPU’s focal plane independent of the objective’s position, as conceptually demonstrated in Figure 1c.

**Figure 2:**
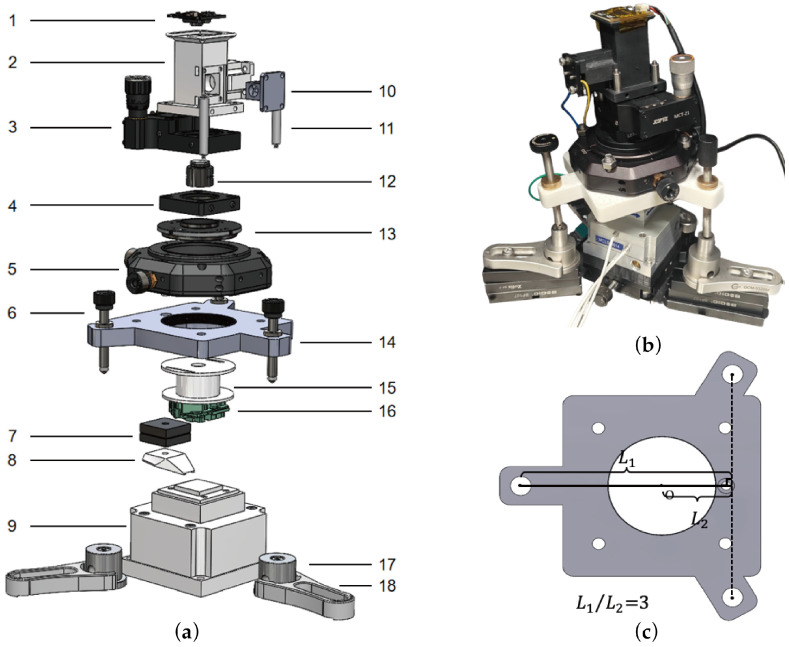
Structure of the AFM system. (**a**) Exploded view of the mechanical structure. (**b**) Photograph of the fully assembled system. (**c**) Top view of the base part (part 14 in (**a**)).

Using this principle, we designed an optical microscope-integrated AFM in this work, as shown in Figure 2. The system adopts a sample-scanning configuration, where the primary scan head is responsible for both the optical measurement and the physical tip approach. The entire scan head and microscopic imaging module are securely mounted on a 3D-printed base supported by three precision lead screws, ensuring both structural simplicity and mechanical rigidity.

The approach process can be precisely executed by rotating the distal lead screw. The base fundamentally acts as a geometric lever mechanism pivoting on the axis formed by the other two support points. In this particular design, the ratio of the actuation lever arm to the probe-end lever arm is L_1_/L_2_ = 3. This implies that the probe displacement is geometrically scaled down to 1/3 of the lead screw displacement, drastically relaxing the requirements for driving precision. In practical operation, manually rotating the distal screw is sufficient to achieve stable and smooth micro-approaches.

The core OPU is securely mounted on a flat, custom x-y translation bracket featuring a 2-inch optical clear aperture. The probe is affixed to a customized holder and magnetically clamped beneath a custom base, facilitating rapid probe installation and exchange. The x-y translation bracket is rigidly positioned above the base. The macroscopic relative position between the OPU and the tip can be roughly adjusted by shifting the retaining ring, followed by precise focusing between the objective lens and the probe tip by actuating the OPU’s VCM. The whole structure is flat and is extremely compact in the z-direction, providing a clear space to integrate the overhead imaging optical path. The corresponding 3D structural models of the above customized components are available in the Supplementary Materials.

The imaging module comprises two identical lenses with a 25 mm focal length (OLD1423-T2M, JCOPTIX, Nanjing, China), configuring a 1:1 relay system that projects the intermediate image from the LD conjugate plane directly onto a CMOS sensor. The system employs a high-power white LED (3 W) for illumination. The emitted illumination beam is reflected by a 50:50 beam splitter and propagates coaxially downward along the OPU’s intrinsic detection optical path, ultimately emerging as parallel light through the OPU objective to perpendicularly illuminate the sample surface.

The entire optical imaging module is constructed using standard 1/2-inch cage system components. The respective lenses are housed in standard lens tubes and mounted on custom optical brackets. The entire assembly interfaces with the lower OPU-tip module via a 1-inch *z*-axis cage translation mount (SM1ZA, THORLABS, Newton, MA, USA), enabling the system to precisely locate the conjugate focal plane. All mechanical linkages are standard off-the-shelf cage components. This construction strategy not only guarantees the reliability and precision of the system but also makes the system open and expandable. Furthermore, the system heavily relies on symmetrical design principles, ensuring highly uniform dimensions and stress distribution around the optical axis, which maximizes overall structural stability and decisively suppresses operational drift. The detailed component parameters and specifications are listed in Table 1.

## 3. Drive and Software Architecture

**Figure 3:**
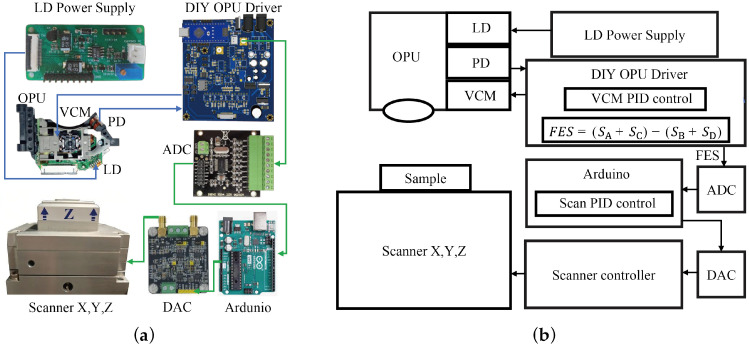
Block diagram of the electromechanical control system. (**a**) Physical wiring diagram. (**b**) System control block diagram.

The electromechanical control architecture, encompassing signal acquisition, feedback control, and motion control, is entirely implemented using standard hardware modules (Figure 3). Because the system strictly operates in contact mode and bypasses high-speed signal modulation and demodulation paradigms, cost-effective mid-tier controllers are perfectly adequate. However, to suppress the intrinsic signal noise of the OPU and concurrently eradicate the VCM’s position drift, we implemented specialized protocols in data acquisition, the custom LD drive circuit, and the software’s scanning logic.

Specifically, to maximize signal fidelity, we employ a 24-bit high-precision analog-to-digital converter (ADS1256, Texas Instruments, Dallas, TX, USA) to sample the FES. The digitized FES is then processed through the scanning PID control logic within the main control unit (Arduino Uno, Arduino S.r.l., Monza, Italy). Finally, a 0~10 V analog control voltage is generated by an off-the-shelf high-precision AD5781 (Analog Devices, Norwood, MA, USA) evaluation board to drive the 3-axis nanopositioning piezoelectric stage (Mad City Labs, Madison, WI, USA) to execute the raster scan. It is worth noting that the commercial nanopositioning stage was utilized here based on laboratory availability; however, alternative low-cost positioning stages can be substituted to further reduce the overall system cost.

Since the OPU’s proprietary built-in drive circuit is closed source, reading raw PD signals and independently driving the VCM is restricted. To bypass this, drawing inspiration from Hwu et al., we developed custom open-architecture OPU drive hardware. It is particularly worth emphasizing that because the original integrated LD driver in the commercial OPU suffers from excessive electrical noise, we completely redesigned a low-noise LD power supply circuit. The laser diode integrates a built-in backlight monitoring photodiode for optical power feedback, which enables automatic power control to maintain a stable laser output. Our design adopts a closed-loop driving scheme that utilizes the internal photodiode to real-time monitor the backward optical power. By dynamically adjusting the driving current through feedback regulation, it effectively suppresses power drift and noise induced by temperature and voltage fluctuations. This custom circuit successfully reduced the LD noise floor by 50%, and that consequently lowers the overall FES noise. The complete schematic diagrams of all relevant circuits in this system are provided in the Supplementary Materials.

**Figure 4:**
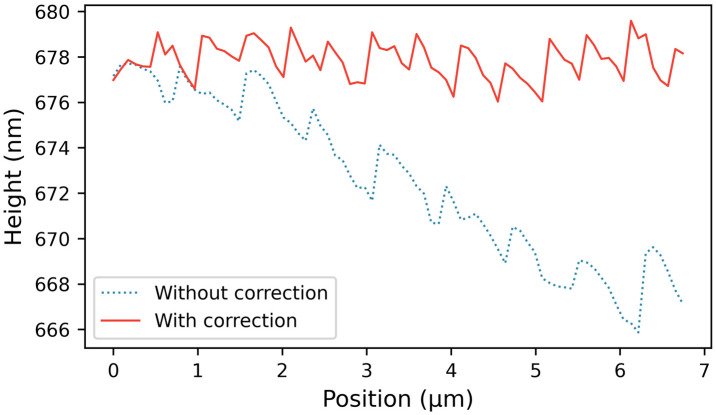
AFM scan line profiles illustrating drift suppression by segmented correction.

Another critical challenge is long-term drift; the VCM exhibits random, unconstrained drift in the z-direction, which directly degrades the AFM’s topographical scanning stability. To suppress this phenomenon, we first applied high-viscosity damping grease to the VCM actuator to improve mechanical stability. Simultaneously, we added a segmented active-correction mechanism into the scanning software loop to combat thermal and mechanical drift during scan processes. Specifically, before initiating each horizontal line scan, the piezoelectric z-stage briefly retracts the sample to break physical contact. During this non-contact window, the baseline FES value is measured, and the VCM is actively adjusted via a secondary feedback loop to force the FES back to absolute zero. This re-zeroing step completely eliminates the accumulated influence of long-term VCM drift and nullifies baseline shifting within individual scan lines. As demonstrated in Figure 4, a typical time-series result on a clean Si wafer substrate reveals that without correction, the system mechanically drifted approximately 15 nm in the z-direction; conversely, with the active correction function enabled, the system’s macroscopic *z*-axis drift is perfectly eliminated, leaving behind strictly the random thermal and electronic noise (RMS noise: 1.1 nm, peak-to-peak noise: 4.1 nm).

## 4. Results and Discussion

**Figure 5:**
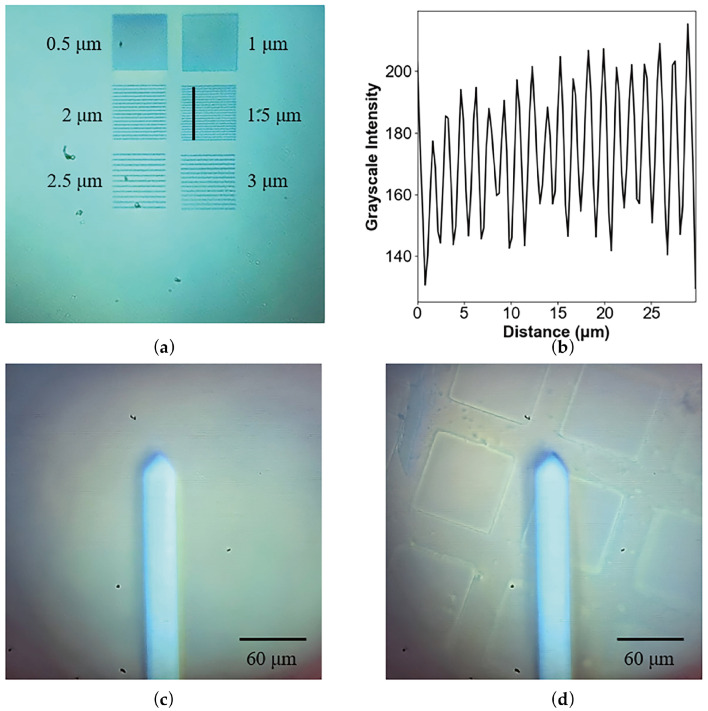
Optical imaging results of the system. (**a**) Imaging of periodic structures. (**b**) Surface profile showing a 1.5 μm period. (**c**) Single-probe imaging. (**d**) Simultaneous imaging of probe and sample.

To evaluate the optical resolution of the system, we fabricated grating structures with various periods (ranging from 0.5 μm to 3.0 μm with an interval of 0.5 μm). Figure 5a shows the optical micrograph, revealing that in the central region, the system’s illumination is uniform without noticeable distortion, and the 1.5 μm grating structure can be clearly resolved. As it possesses inherent PID feedback capabilities linked to the FES, this system can also function as a standalone optical microscope with automatic real-time autofocus when the AFM probe is removed.

With the help of the optical imaging capability, we are able to monitor the tip’s condition throughout AFM experiments, as illustrated in Figure 5c,d. It is important to note that when the AFM cantilever is inserted into the optical path, its highly reflective metallic coating inevitably degrades the sample’s optical imaging contrast. Nevertheless, despite this contrast reduction, the system reliably resolves micron-scale morphological features on the sample surface. This capability is crucial for visually determining the precise macroscopic relative position between the sharp probe tip and the sample features, paving the way for advanced functions like automated region-of-interest (ROI) selection and large-area image stitching.

**Figure 6:**
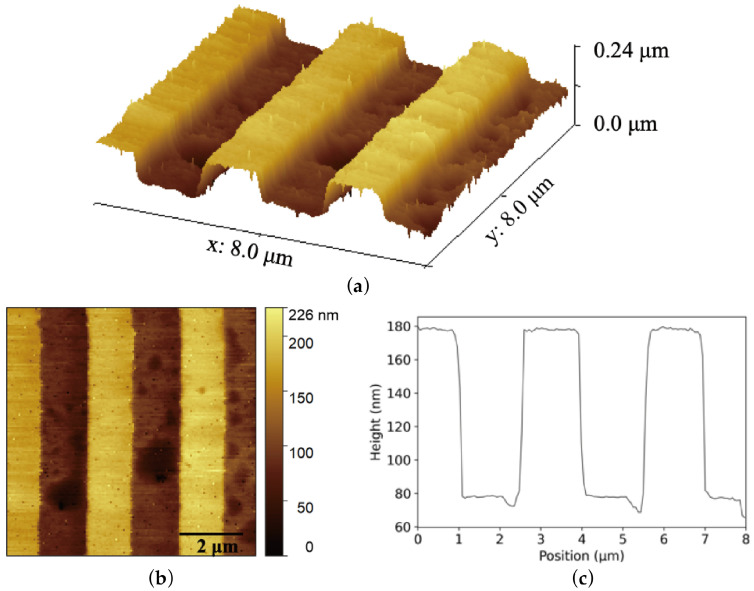
AFM scanning results. (**a**) 3D height map representation of the topography. (**b**) Topography image of a calibration step grating sample. (**c**) Cross-sectional profile of the topography image.

Using the optimized system, we achieved stable topographical scanning in contact mode at a line scan rate of 0.5 Hz. This line scan rate is mainly restricted by the processing capacity of the Arduino control platform, such as its analog sampling bandwidth, serial communication speed, and closed-loop control iteration period. Figure 6 presents a representative scan result of a 1D periodic calibration step grating (period: 3 μm, step height: 107 nm). The extracted cross-sectional profile vividly demonstrates exceptional flatness across the scan lines, with random topographical noise maintained well below 5 nm. This impressive metric aligns perfectly with the noise floor recorded in Figure 4, confirming that our low-cost DIY system effectively approaches the performance baseline of commercial AFMs. All AFM topography images and cross-sectional profiles in this work were processed using the open-source software Gwyddion 3.7 [25].

The integration of real-time optical imaging and stable contact-mode scanning addresses key operational drawbacks commonly encountered in low-cost OPU-based AFM platforms. Compared with single-function open-source alternatives, the simultaneous optical feedback reduces the uncertainty of tip-sample positioning and helps minimize unintended probe–sample collisions during adjustment and scanning. The implementation of modular commercial components and simplified 3D-printed structures improves the repeatability and maintainability of the system, which is beneficial for large-scale replication in multi-user environments.

It should be objectively noted that while our software-based segmented correction mechanism elegantly eliminates cumulative signal drift caused by the VCM, the periodic retract-and-measure cycle inevitably compromises the maximum scanning speed. To permanently eradicate this bottleneck in future iterations, the VCM must be entirely bypassed and replaced with a mechanically rigid *z*-axis adjustment paradigm, such as a precision z-translation stage driven by a micro-stepper motor.

## 5. Conclusions

To realize a high-performance, reliable, and user-friendly low-cost AFM tailored for DIY and educational communities, this paper developed a novel OPU-based AFM system seamlessly integrated with real-time optical microscopy. By targeting the chronic issues of optical monitoring difficulty, severe mechanical drift, and high signal noise inherent in traditional OPU-AFMs, we implemented specific engineering solutions across the optical, hardware, and software domains. By creatively leveraging an integrated conjugate-plane imaging design, we successfully solved the challenge of the simultaneous real-time optical monitoring of both the probe and the sample. By completely overhauling the LD module’s drive circuitry, the system’s baseline noise floor was considerably suppressed. Furthermore, we introduced a dynamic segmented calibration mechanism, which can eliminate the long-term thermal and positional drift of the VCM coil, making sustained and stable contact-mode scanning a reality. Built upon standard commercial optomechanical components, the system not only guarantees structural stability and measurement reliability but also inherently possesses good compatibility and expandability, offering a robust foundation for subsequent design optimizations and advanced functional extensions.

## Figures and Tables

**Table 1:** List of parts of the system.

Part No.	Name	Model	Cost (USD)
1	CMOS camera	AILICHENG ELECTRONICS, ALC-0V5693	12.57
2	Camera mount	3D printed	0.98
3	*Z*-axis translation stage	THORLABS, SM1ZA	235.61
4	30 mm cage plate with SM1 thread	THORLABS, CP33	20.43
5	60 mm cage system translating lens mount for 2-inch optics	THORLABS, CXY2	304.95
6	Fine adjustment screw (3 pieces)	THORLABS, FAS300	35.76
7	1-inch kinematic mount	THORLABS, KB1 × 1	86.79
8	Tip holder	3D printed	0.11
9	Piezo stage	MAD CITY LABS, CUSTOM 3AXIS NANOPOSITIONING SYSTEM	~20,000
10	Beamsplitter holder	3D printed	0.08
11	Post (4 pieces)	THORLABS, ER2	28.2
12	1/2-inch lens tube	JCOPTIX, SM05L12-A	10.7
13	adaptor	THORLABS, SM1A71	33.99
14	Base	3D printed (Formlabs Rigid 10 K)	17.65
15	OPU-mount	3D printed	0.47
16	OPU	SANYO, SF-HD850	2.34
17	1-inch pedestal pillar post (3 pieces)	THORLABS, RS1P4M	81.99
18	Clamping fork (3 pieces)	THORLABS, CF125	31.08

3D printed parts are self-fabricated, and their costs are calculated based on material expenses.

## Data Availability

The raw data supporting the conclusions of this article will be made available by the authors on request.

## Supplementary Materials

The following supporting information can be downloaded at https://www.mdpi.com/article/10.3390/s26103170/s1. Schematic_Diagram_Overall_Circuit.pdf: Complete circuit schematic of the system; Base.step: 3D model of the base component; Beamsplitter holder.step: 3D model of the beamsplitter holder; Camera mount.step: 3D model of the camera mount; OPU-mount.step: 3D model of the OPU mount; Tip holder.step: 3D model of the tip holder.
