# Supplementary figures and images for: Low-Cost Integrated Optical Microscope and Contact-Mode Atomic Force Microscope System Based on DVD Optical Pickup Unit
Source: Sensors (Basel). 2026 May 17;26(10):3170. doi: 10.3390/s26103170 (PMC13210909; doi:10.3390/s26103170)

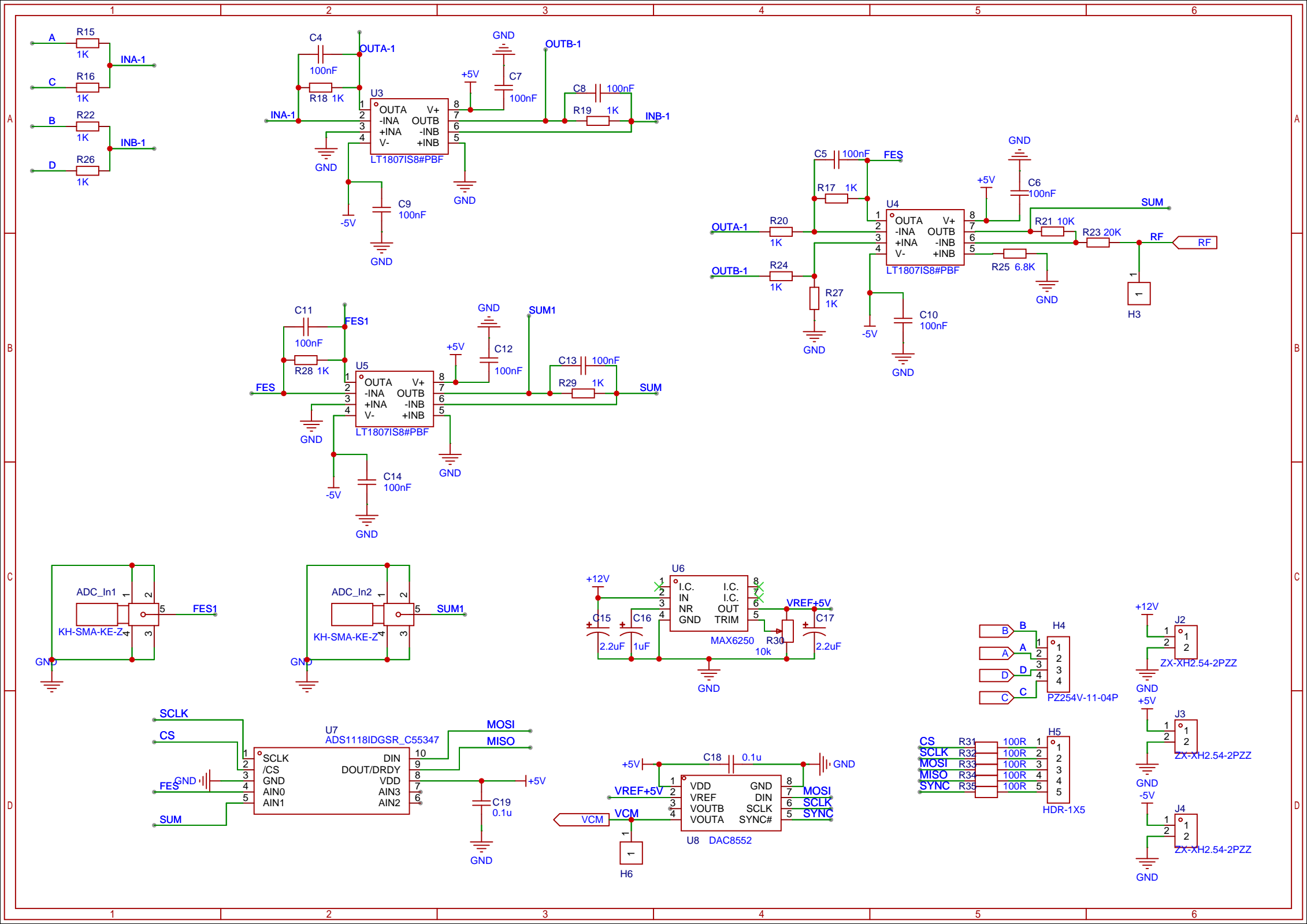

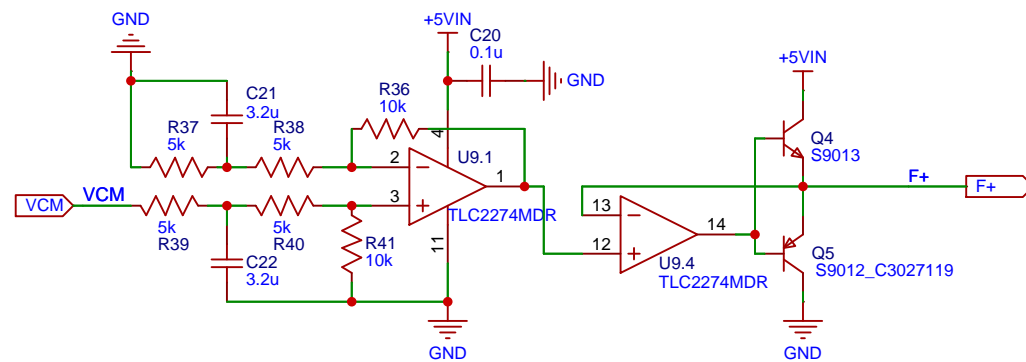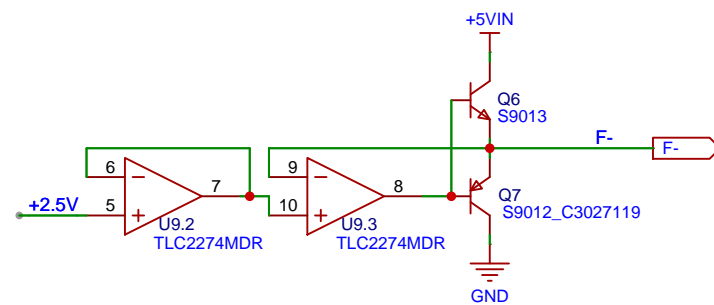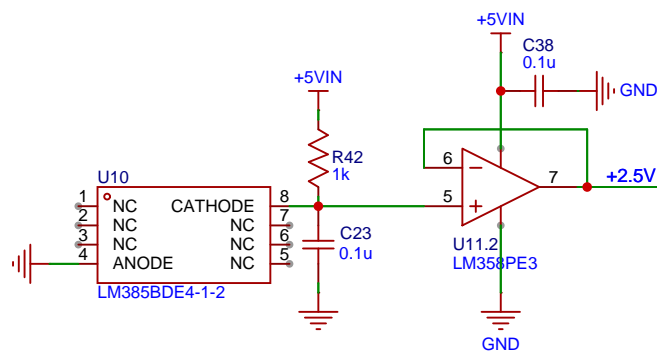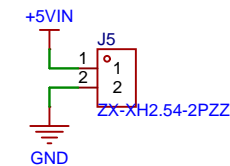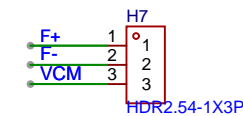

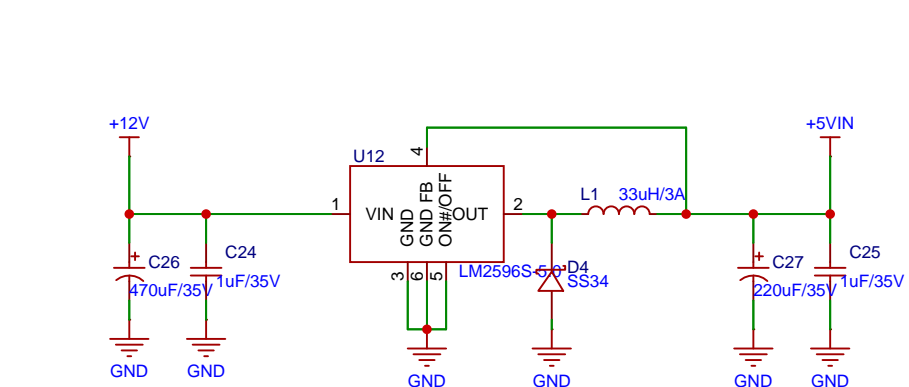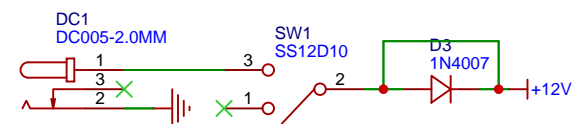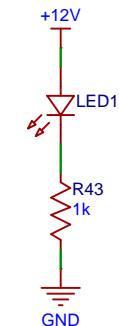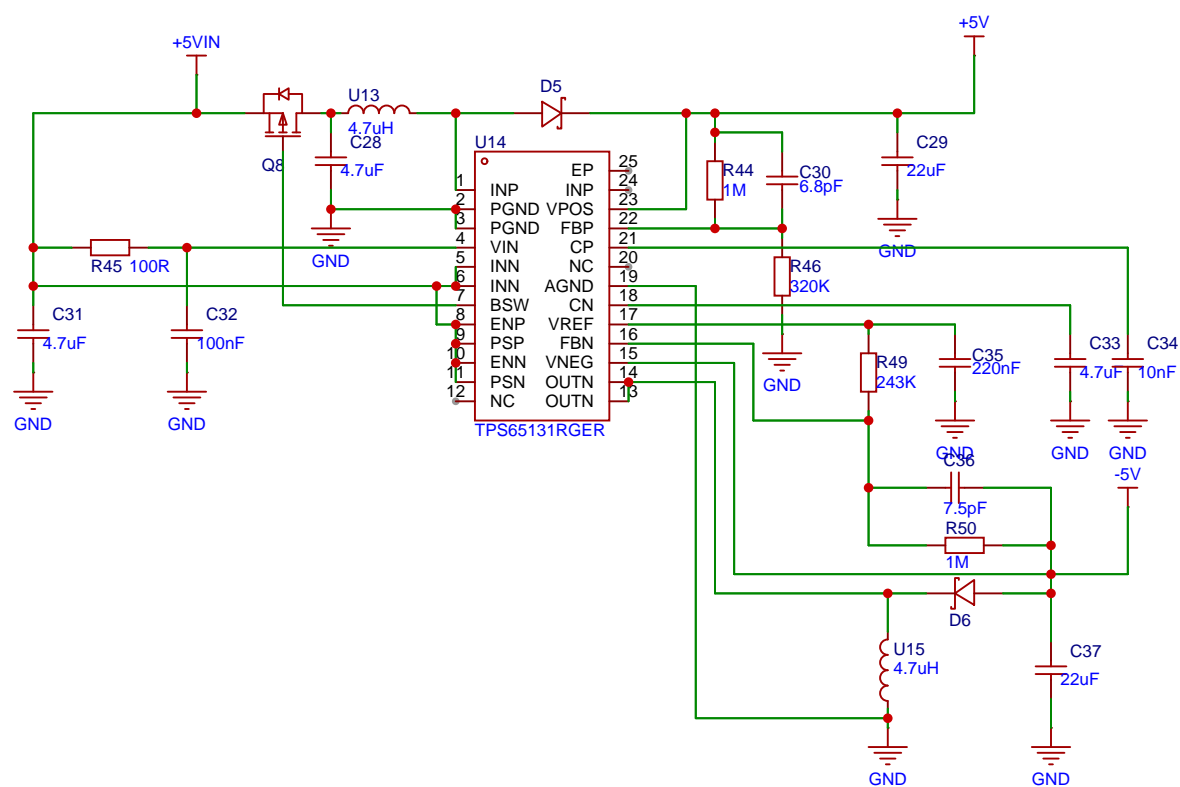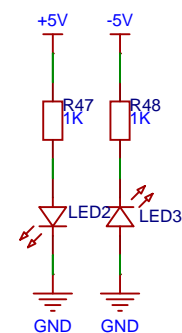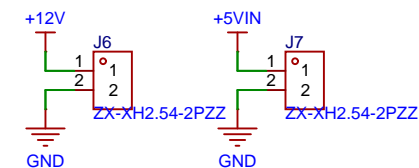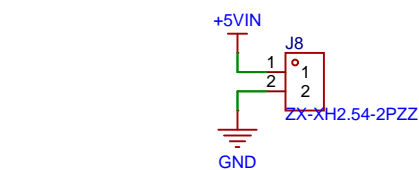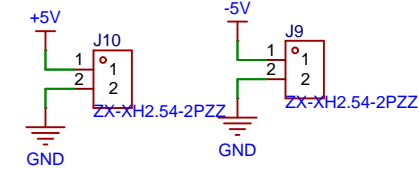

Supplement: Supplementary file 1 [file sensors-26-03170-s001.zip › Schematic_Diagram_Overall_Circuit.pdf]
